# Monthly Summary

**Published:** 1881-11

**Authors:** 


### MONTHLY SUMMARY.

---

*Women as Physicians.*—Saratoga, N. Y., September 7.—At the session of the Social Science Congress, Dr. Emily Pope read a paper on "The Practice of Medicine by Women in the United States." The objects were to show to what extent women were practising medicine in this country; whether the majority of women graduates in medicine devote themselves to its practice; how far their pecuniary success shows a demand on the part of the public for educated women physicians; what effect the strain of practice has upon their health; what proportion of them marry, and with what results to their professional career. The 470 circulars sent out to women physicians have brought statistics showing that 390 are engaged in active practice, 11 never practiced, 29 have retired after practicing, 12 of whom after marriage, 7 retired from ill health, and 5 have taken up other work. These women are in 26 States, New York, Massachusetts and Pennsylvania having the largest proportion. Of those heard from 75 per cent. were single when they began the study, 19 per cent were married, and 6 per cent. widows. Average age when they began the study, 27 years; 144 practiced less than 6 years, 123 between 5 and 10 years, 40 from 10 to 15 years, 15 from 15 to 20 years, 23 over 20 years; 341 practiced regular medicine, 13 homœopathy, 10 gave no answers, 77 report that they supported themselves from the beginning, 138 say their incomes are still insufficient.

---

*Death from Nitrous Oxide.*—A death from inhalation of nitrous oxide gas occurred lately at Exeter, England. The gas was given to produce insensibility during the extraction of a tooth. The patient was a woman of about forty years of age. After a few inhalations, the pulse was noticed to become weak, and the administration was stopped for a time. As the patient had not become insensible, the inhalation was resumed, and the tooth withdrawn. The patient had become livid, and in a few minutes died. Her health had been excellent previously, and there was no reason known why she would not be a good subject for the gas. A case has also been reported in this country. —*Druggists Circular.*

